# Phytochemical Composition, Antibacterial Activity, and Antioxidant Properties of the *Artocarpus altilis* Fruits to Promote Their Consumption in the Comoros Islands as Potential Health-Promoting Food or a Source of Bioactive Molecules for the Food Industry

**DOI:** 10.3390/foods10092136

**Published:** 2021-09-09

**Authors:** Toilibou Soifoini, Dario Donno, Victor Jeannoda, Danielle Doll Rakoto, Ahmed Msahazi, Saidi Mohamed Mkandzile Farhat, Mouandhoime Zahahe Oulam, Gabriele Loris Beccaro

**Affiliations:** 1 Laboratoire Aliments, Réactivité et Synthèse des Substances Naturelles, Faculté des Sciences et Techniques, Université des Comores, Moroni 167, Comoros; toilibousoifoini@yahoo.fr (T.S.); archiam2017@gmail.com (A.M.); 2 Dipartimento di Scienze Agrarie, Forestali e Alimentari (DISAFA), Università degli Studi di Torino, 10095 Grugliasco, Italy; gabriele.beccaro@unito.it; 3 Laboratoire de Biochimie Appliquée Aux Sciences Médicales-Faculté des Sciences, Université d’Antananarivo, Antananarivo 101, Madagascar; victor_jeannoda@yahoo.fr (V.J.); dad.rakoto@yahoo.fr (D.D.R.); 4 Faculty of Medicine, University of Sherbrooke, Campus Longueuil, Longueuil, QC J4K 0A8, Canada; farhat@live.ca; 5 Département de Chimie, Biochimie et Physique, Université du Québec à Trois-Rivières, Trois-Rivières, QC G8Z 4M3, Canada; zahahe.oulame.mouandhoime@uqtr.ca

**Keywords:** breadfruit, bioactive molecules, antibacterial properties, traditional food, Comoros, antioxidants

## Abstract

The present study aimed to evaluate the health-promoting potential of breadfruit (*Artocarpus altilis* (Parkinson) Fosberg, Moraceae family), a traditional Comorian food, considering the sample variability according to geographic localisation. Moreover, the main aims of this research were also to promote its consumption in the Comoros Islands as potential health-promoting food and evaluate it as a source of bioactive molecules for the food industry thanks to its antioxidant and antibacterial properties. Investigations on biologically active substances were carried out on the extracts obtained from breadfruit flours from five regions of Grande Comore (Ngazidja), the main island in Comoros. Phytochemical screening revealed the presence of tannins and polyphenols, flavonoids, leucoanthocyanins, steroids, and triterpenes. The considered secondary metabolites were phenolic compounds, vitamin C, monoterpenes, and organic acids. The contents of total phenolic compounds (mgGAE/100 g of dry weight—DW) in the extracts ranged from 29.69 ± 1.40 (breadfruit from Mbadjini—ExMBA) to 96.14 ± 2.07 (breadfruit from Itsandra—ExITS). These compounds included flavanols, flavonols, cinnamic acid and benzoic acid derivatives, and tannins which were detected at different levels in the different extracts. Chlorogenic acid presented the highest levels between 26.57 ± 0.31 mg/100 g DW (ExMIT) and 43.80 ± 5.43 mg/100 g DW (ExMBA). Quercetin was by far the most quantitatively important flavonol with levels ranging from 14.68 ± 0.19 mg/100 g DW (ExMIT) to 29.60 ± 0.28 mg/100 g DW (ExITS). The extracts were also rich in organic acids and monoterpenes. Quinic acid with contents ranging from 77.25 ± 6.04 mg/100 g DW (ExMBA) to 658.56 ± 0.25 mg/100 g DW of ExHAM was the most important organic acid in all the breadfruit extracts, while limonene was quantitatively the main monoterpene with contents between 85.86 ± 0.23 mg/100 g DW (ExMIT) and 565.45 ± 0.24 mg/100 g DW (ExITS). The antibacterial activity of the extracts was evaluated on twelve pathogens including six Gram (+) bacteria and six Gram (−) bacteria. By the solid medium disc method, except for *Escherichia coli* and *Pseudomonas aeruginosa*, all the bacteria were sensitive to one or more extracts. Inhibitory Halo Diameters (IHDs) ranged from 8 mm to 16 mm. *Salmonella enterica*, *Clostridium perfringens*, and *Vibrio fischeri* were the most sensitive with IHD > 14 mm for ExITS. By the liquid microdilution method, MICs ranged from 3.12 mg/mL to 50 mg/mL and varied depending on the extract. *Bacillus megaterium* was the most sensitive with MICs ≤ 12.5 mg/mL. *Pseudomonas aeruginosa*, *Shigella flexneri*, and *Vibrio fischeri* were the least sensitive with all MICs ≥ 12.5 mg/mL. ExHAM was most effective with a MIC of 3.12 mg/mL on *Staphylococcus aureus* and 6.25 mg/mL on *Salmonella enterica*. The antioxidant power of the extracts was evaluated by the FRAP method. The activity ranged from 5.44 ± 0.35 (ExMBA) to 14.83 ± 0.11 mmol Fe^2+^/kg DW (ExHAM). Breadfruit from different regions of Comoros contained different classes of secondary metabolites well known for their important pharmacological properties. The results of this study on phenolics, monoterpenes, and organic acids have provided new data on these fruits. The obtained results showed that breadfruit from the biggest island of the Union of Comoros also presented antimicrobial and antioxidant properties, even if some differences in effectiveness existed between fruits from different regions.

## 1. Introduction

Malnutrition is a public health problem in developing countries [1,2]. In the Comoros Islands, as in many countries of Southern Africa, malnutrition and food insecurity affect a very large percentage of the population [1,3,4]. Food security is ensured when all human beings have, always, the physical, social, and economic possibility of obtaining sufficient, healthy, and high-nutritional food to enable them to meet their food needs and preferences to lead a healthy and active life. Currently, despite the Comorian government efforts to fight famine and undernourishment, malnutrition is still one of the main causes of death in children aged 0 to 5 [5].

Food insecurity in Comoros shows a very worrying level due to poverty. The Global Hunger Index (GHI), assessed by the International Food Policy Research Institute (IFPRI), showed an increase of almost 17%, placing the Comoros Islands in 73rd place out of 81 surveyed countries. IFPRI statistics specified that 46% of Comorians are undernourished and under-5 children, whose mortality rate is estimated at 10.4% with 22% of cases of death, are underweight [6]. Overall, 30% of these children suffer from chronic malnutrition and 15% in severe form. About one in ten children is acutely malnourished and 4% in the severe form; in 15% of cases, children are underweight [7,8]. Agricultural development should play a leading role in alleviating world hunger and increasing global food security. The high rate of malnutrition and the geographical isolation of the three islands of the Comorian archipelago, where air and sea services are extremely limited, confirms the need to increase local food production [4].

Breadfruit (*Artocarpus altilis* (Parkinson) Fosberg) is a traditional food crop cultivated for its starchy fruits throughout Oceania [9]. Breadfruit production yields of 6 t/ha (edible dry weight) have been reported [10]. This is an impressive yield compared to current staple crops, with average yields of about 5 t/ha for rice (2019), 8 t/ha for maize (2019), and 3.5 t/ha for wheat (2019) [11]. In Africa, and particularly in the Union of Comoros, breadfruit surprisingly remained neglected for many years despite its strong nutritional and medicinal potential. Research on the chemical constituents of breadfruit has isolated several classes of compounds such as various triterpenes and flavonoids. *Artocarpus altilis* is a rich source of prenylated phenolic compounds such as geranylated flavones. The pharmacological studies have indicated that some flavonoids from breadfruit (*A. altilis*) have anti-inflammatory activities and can inhibit 5-lipoxygenase of cultured mastocytoma cells, cathepsin K, and 5α-reductase [9]. Breadfruit is consumed primarily for its nutritional benefits and as a major source of carbohydrates. Fruits and seeds are good sources of carbohydrates, protein, dietary fiber, fatty acids, pro-vitamin A, potassium, and calcium with significant amounts of ascorbic acid, niacin, and iron [10].

The country presents a significant agrobiodiversity of natural food resources, unfortunately underutilized or neglected, which could solve, at least in part, the problems of food insecurity and malnutrition. This study was mainly aimed to evaluate the potentialities of the fruits of *Artocarpus altilis* from Grande Comore to promote and better understand the contribution of these natural plant resources to the improvement of the nutritional and socio-economic conditions of the local populations. This research aimed to determine the phytochemical composition, antibacterial activity, and antioxidant properties of *Artocarpus altilis* fruits to promote their consumption in Comoros, as a potential health-promoting food.

## 2. Materials and Methods

### 2.1. Study Area and Plant Materials

The climate of the study area (Grande Comore) is generally mild, humid, and tropical, and the two main seasons present different raininess (in total, about 2700 mm per year). It is subjected to three successive regimes of wind: (i) the north-west monsoon/trade winds or “Kashkazi”, (ii) local winds from the south-west originating from the southern high pressures, and (iii) the south-east monsoon/trade winds or “Kusi”. The country is vulnerable to climate change. Despite the presence of two seasons, the average temperature varies little throughout the year; indeed, the temperature reaches an average of 30 °C in March, the hottest month in the rainy season (from November to April), and an average of 20 °C in the cool-dry season (from May to October). This island is rarely subject to cyclones.

Although all islands are of volcanic origin, Comoros has morphological characteristics and soil types that vary depending on the age of volcanism. The island of Grande Comore consists of two shield volcanoes, one of which has gone through several phases of activity during the twentieth century (“Karthala”). There are no permanent water systems in Grande Comore because of the high permeability of the soils.

Pedoclimatic conditions, such as volcanic soil, high temperatures, and well-distributed rains (even if the rainfall is heavier in summer than in winter), influence bioactive compound content in fresh fruits. 

**Figure 1:**
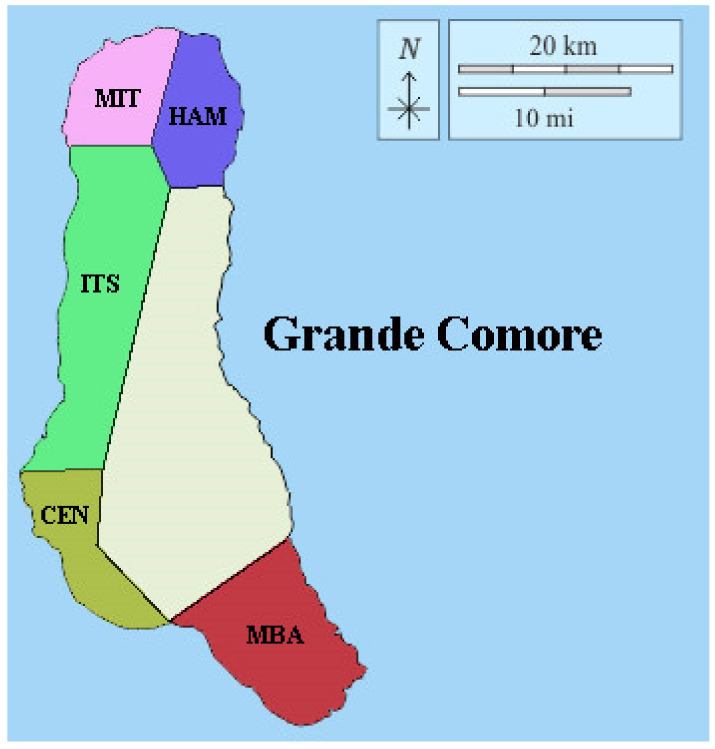
Breadfruit collection regions in *Grande Comore*. MIT: Région of Mitsamihuli; HAM: région of Hamahamet; ITS: région of Itsandra; CEN: Region of Centre; MBA: Region of Mbadjini.

Ten fruits of breadfruit (*Artocarpus altilis* (Parkinson) Fosberg, *Moraceae* family) were randomly selected and harvested at the commercial maturity stage from three plants for each biological replication (n = 3) for each different region of the Union of Comoros (Figure 1) and sun-dried (temperature ranges: from 25 °C to 35 °C) for about 3 days. 

The analysed samples are designated as shown in Table 1.

### 2.2. Solvents and Chemical Products

They were purchased from different suppliers:

- Sigma-Aldrich (St. Louis, MO, USA) for sodium carbonate, Folin-Ciocalteu phenol reagent, sodium acetate, citric acid, potassium chloride, hydrochloric acid, iron chloride (III) hexahydrate, 2,4,6-tripyridyl-S-triazine and 1,2-phenylenediamine dihydrochloride;
- Sigma-Aldrich (St. Louis, MO, USA) for all polyphenolic and terpenic standards, potassium dihydrogen phosphate, phosphoric acid, methanol, and HPLC grade acetonitrile;
- Fluka Biochemika (Buchs, Switzerland) for acetic acid, ethanol, organic acids, and HPLC-grade formic acid;
- AMRESCO (Solon, OH, USA) for the disodium salt of ethylene diamine tetra-acetic acid;
- Riedel-de Haen (Seelze, Germany) for sodium fluoride;
- Extra-synthesis (Genay, France) for cetyltrimethylammonium bromide (cetrimide), ascorbic acid (AA), and dehydroascorbic acid (DHAA);
- Sartorius Stedim Biotech (Arium, Göettingen, Germany) for the ultra-pure Milli-Q water.

### 2.3. Phytochemical Screening

The extracts prepared from the dried fruit powder were screened for phytochemical constituents (alkaloids, saponins, flavonoids, tannins, polyphenols, iridoids, leucoanthocyanins, steroids, and triterpenes) using simple qualitative methods [12,13,14,15].

### 2.4. Preparation of Extracts for Spectrophotometric and Chromatographic Analysis

After removing the superficial green portion, the fruits were cut into halves. The heart was removed, and the remaining portion was peeled into pieces (each fruit piece is quite ellipsoidal, about 70 mm in length and 30 mm in width, with a weight of about 25 g) as performed by the local population. These little pieces were subsequently dried. Pieces of 3-day sundried breadfruits were ground with a ceramic mortar (size: 10 mm × 10 mm) and milled with an automatic grinder to be reduced in powder size. About 10 g of breadfruit flour are weighed. The extraction solvent consisted of a mixture of methanol, water, and HCl (95:4.7:0.3, *v*/*v*/*v*) [16]. For each sample, 50 mL of solvent were required for extraction. The mixture was macerated in the dark for 72 h, with magnetic stirrings from 5 to 10 min per day. The mixture was filtered, and the filtrate was retained. A second extraction was performed on the marks with another 50 mL of extraction solution. The solvent-marc mixture was treated as the previous extraction and filtered on paper. The marcs were manually pressed to obtain the maximum filtering. The extracts were again filtered. The obtained filtrate was added to the first one and stored until analysis under normal conditions at 4 °C and 95% relative humidity [17]. It should be noted that all the manipulations were repeated three times.

### 2.5. Determination of the Total Polyphenolic Content (TPC)

The method used for the determination of the total polyphenol composition is based on the reaction of Folin-Ciocalteu. The used reagent consists of a mixture of phosphotungstic acid and phosphomolybdic acid which is reduced when phenols are oxidised to a mixture of tungsten blue oxide and molybdenum [18]. For polyphenol quantification, 30 mL of distilled water, 2.5 mL of Folin Ciocalteu reagent (Sigma-Aldrich, Germany), and 10 mL of 15% sodium bicarbonate solution are added in 500 µL of the methanolic extract [19,20,21]. Volume is adjusted to 50 mL with distilled water, and the reading of the optical density is read at 750 nm with a spectrophotometer. The same procedure is applied to the blank, but the extract is replaced by the extraction solvent [22]. Results are expressed in mg gallic acid equivalent (GAE) per 100 g dried weight (DW) [16,23].

### 2.6. Chromatographic Analysis

This technique allows the analysis and determination of phytochemical compounds present in plant extracts. An Agilent 1100 HPLC system (Agilent 1200, Santa Clara, CA, USA) equipped with a G1311A quaternary pump, a manual injection valve, and a 20 μL sample loop coupled with an Agilent GI315D UV-Vis diode array detector were used for the analysis. All the substances were identified by comparison and combination of their retention times and UV-vis spectra with standards under identical chromatographic conditions. The external standard method was then used for quantitative determination. For this, calibration curves with a concentration of 125 to 1000 mg/L were produced [24]. All the chromatographic methods are described in Table S1 (Supplementary Materials).

#### 2.6.1. Quantitative Determination of Polyphenols

For the analysis of polyphenols, filtration is necessary to separate phenolics and vitamin C. The step started with the activation of the SPE filter (C18 cartridge, Sep-Pak C-18), rinsing it successively with 5 mL of methanol and 5 mL of distilled water with a syringe. Two millilitres of extract were recovered by a syringe and injected through the dried filter to remove vitamin C. Polyphenols were retained by the filter. Two ml of methanol were injected again to recover the polyphenols retained on the filter. Samples were stored at a temperature of 4 °C until the HPLC analysis.

##### Conditions for the Analysis of Cinnamic Acid and Flavonols

Two mobile phases were used for the analysis of cinnamic acids and flavonols by HPLC (Agilent 1200, Santa Clara, CA, USA). Mobile phase A was acetonitrile. The second mobile phase B was water containing 10 mM potassium phosphate (KH_2_PO_4_). Elution was performed by a gradient. It was carried out with a flow rate of 1.5 mL per minute and a duration of 20 min. Cinnamic acids and flavonols were detected at 330 nm. 

##### Conditions for the Analysis of Benzoic Acids, Catechins, and Tannins

Two mobile phases were used. The first consisted of a solution of methanol-water—formic acid (5:95:0.1; *v*/*v*/*v*) and the second of a mixture of methanol—formic acid (100:0.1; *v*/*v*). Analysis was carried out by gradient with a flow rate of 1 mL/min for 35 min. Benzoic acids, catechins, and tannins were detected at 250, 280, and 320 nm, respectively.

#### 2.6.2. Quantitative Determination of Organic Acids

For organic acids, extracts were directly analysed by HPLC. Two mobile phases were used for this analysis. The first phase was an aqueous solution of potassium phosphate KH_2_PO_4_ whose pH has been adjusted to 2.8 with phosphoric acid. The second mobile phase was acetonitrile. Isocratic analysis was performed. The flow rate was 0.5 mL/min, and the analysis time was 20 min. Organic acids were read at 214 nm. 

#### 2.6.3. Quantitative Determination of Monoterpenes

Extracts were analysed directly after extraction. Water and methanol were the two mobile phases used. Analysis was performed by gradient with a flow rate of 1 mL/min and a duration of 75 min. Monoterpenes were detected at 220 and 235 nm.

#### 2.6.4. Quantitative Determination of Vitamin C

Two ml of methanolic extract were centrifuged at 12,000 rpm for 5 min at 4 °C to obtain a homogeneous extract. The previously obtained extract was filtered on a 0.45 μm diameter filter (Titan 2 HPLC filter 17 mm PTFE Membrane). The SPE filter (C18 cartridge, Sep-Pak C-18, Waters Corporation, Milford, MA, USA) separated the polyphenol from vitamin C in each extract. The filter was rinsed successively with 5 mL of methanol and 5 mL of distilled water with a syringe. After drying the filter, 2 mL of each extract were recovered by a syringe and injected into the filter. The polyphenols were retained on the filter, but vitamin C passed through the filtrate and was recovered in a 2 mL tube and stored at 4 °C. 

Analysis of vitamin C from the extracts by HPLC required specific treatment to separate it into ascorbic acid and dehydroascorbic acid [24,25]. The filtered sample (750 μL) and the specific reagent (OPDA—o-Phenylenediamine) for the separation of ascorbic acid and dehydroascorbic acid (250 μL) were added into a 2 mL test tube. The mixture was placed in the dark at 4 °C. After 30 min, separation time, 20 μL of the sample were injected into the HPLC with a syringe. Note that reagents for vitamin C separation were prepared on the analysis day. Agitation before use and storage at 4 °C in the dark were recommended.

Only one mobile phase was used for the analysis of vitamin C by HPLC. It consisted of 50 mm potassium phosphate and 5 mm cetrimide in a hydro-methanol solution (5:95, *v*/*v*). Analysis was carried out at a flow rate of 0.9 mL per min for 30 min. Vitamin C was detected at 261 nm and 348 nm. 

### 2.7. Antibacterial Activity

The methanolic extracts (ExMIT, ExHAM, ExITS, ExCEN, and ExMBA) were used. Twelve bacteria involved in human pathologies including six Gram-positive and six Gram-negative were tested (Table 2). They come from the collection of the Laboratory of Biochemistry Applied to Medical Sciences (LABASM). The used media were at the quality for analysis and BIORAD brand:

- MUELLER-HINTON Agar (MHA) medium to study the microorganism sensitivity for extracts in a solid medium;
- MUELLER-HINTON (MHB) broth to study the extract activity in a liquid medium. Ready-to-use imipenem impregnated disks (10 µg) were used as a reference.

#### 2.7.1. Evaluation of Antibacterial Activity by the Solid Medium Diffusion Method (Antibiogram Test)

Each bacterial strain was subcultured in MHA agar medium in Petri dishes according to the exhaustion method, then incubated in an oven according to conditions of temperature and optimal duration of the culture of each bacteria, to obtain a young culture and isolated colonies.

From the previous cultures, few isolated colonies were suspended in physiological water. The turbidity of this suspension was adjusted to that of the 0.5 Mac Farland standard and then diluted to 1/100 to obtain an inoculum estimated at 106 cells/mL. This inoculum was inoculated by flooding onto Petri dishes containing MHA agar.

Sterilised antibiogram discs (6 mm in diameter), pre-impregnated with 10 μL of extract to be tested (1 mg/disc), were delicately placed on the surface of the inoculated agar. This concentration, often used in the evaluation of the antibacterial activity of plants [26,27,28,29], is also used at LABASM [30]. The extract diffused from the disc creates a concentration gradient. Antibacterial activity was indicated by the presence of an inhibitory halo around the disc. The larger the inhibition halo, the more sensitive the microorganism. The experiments were performed in triplicate.

The diameters of the inhibitory halos or IHD (mm) were measured after 24 h, and the results were expressed according to the standards indicated in Table 3 [31,32].

#### 2.7.2. Determination of Minimum Inhibitory Concentration (MIC) and Minimum Bactericidal Concentration (CMB)

MIC is the lowest concentration of antibiotic that gives growth inhibition. This concentration was determined for active extracts on tested microorganisms (IHD greater than or equal to 9 mm) according to the method of dilution in liquid medium on a microplate used by Andriamampianina et al. (2016) [30]. This is a double dilution method.

One year of bacteria taken from a preculture was adjusted to 0.5 Mac Farland and reduced to 106 cells/mL in Mueller-Hinton broth (MHB). The inoculum was then obtained. A cascade dilution of extracts sterilised by filtration (Sartorius Stedim Biotech 0.2 μm) was carried out to obtain a range of precise concentrations. Two controls were used: a negative control (T− or no growth) containing 100 μL of MHB and a positive (T + or growth) (5 μL of inoculum and 95 μL of MHB).

A volume of 100 μL of each extract dilution was transferred to the wells of the microplate. Plates were then covered with sterile aluminum foil, then incubated at 37 °C. After incubation, 40 μL of para-iodonitrotetrazolium chloride (INT) solution at a concentration of 0.2 mg/mL were added to each well. INT is a coloured indicator that is yellow and turns purple when microbial growth occurs. The plate was incubated again in the same way as before. MIC corresponds to the lowest concentration of the tested extract showing no change in colour [30]. To determine CMB, 5 μL of each well, which do not show any purple colouration, were subcultured onto MHA medium. CMB is the lowest concentration at which no bacterial colony grows after incubation.

According to Michelle da Silva (2013) [33], there is no consensus regarding the antibacterial activity of natural products. The CMB/MIC ratio indicates the nature of the effect of the extract on micro-organisms. When this ratio is greater than 4, the effect is bacteriostatic, and if it is less than or equal to 4, the effect is bactericidal [34].

### 2.8. Antioxidant Capacity

The same extracts used for the study of antibacterial activity were tested. The antioxidant capacity of breadfruit flour was evaluated by the FRAP method (Ferric Reducing Antioxidant Power). This method was based on the reduction of the ferric ion (Fe^3+^) in the solution of di 2,4,6-Tripiridil-S-Triazine (TPTZ) to a ferrous ion (Fe^2+^) [35,36]. Samples and blank were placed in a 37 °C water bath for 30 min. Optical density was read using a UV/Visible spectrophotometer (1600-PC, VWR International, Radnor, PA, USA) at 595 nm [24]. Results were expressed in millimoles of Fe^2+^ equivalents per kg DW [22].

### 2.9. Statistical Analysis

Mean values and the deviation standard (SD) are analysed by the T-Student and ANOVA test of the various extracts to define significant differences between the different samples of breadfruit flours. The *p* < 0.05 differences were considered statistically significant. The results were expressed as mean values with relative deviation standards (SD).

## 3. Results and Discussion

### 3.1. Phytochemical Screening and Total Polyphenolic Content

All the extracts contained the same secondary metabolites: deoxyosis, tannins and polyphenols, leucoanthocyanins, flavonoids, steroids, and triterpenes. Sometimes they were present in varying amounts depending on the extract. Alkaloids, saponins, and iridoids were not detected in all the extracts. All five extracts contained the same chemical groups as reported in the extracts from Malaysian breadfruit [37].

The main secondary metabolites identified in the extracts of breadfruit from five regions are presented in Table 4.

Polyphenol content was significantly (*p* < 0.05) different in the five extracts. The highest value was detected in ExITS (96.14 ± 2.07 mgGAE/100 g dry matter, DW) and the lowest in ExMBA (29.69 ± 1.40 mgGAE/100 g DW), similarly to other common fruits (e.g., apples and kiwi with a range of 25–75 mgGAE/100 g) as reported in previous studies [16,17]. Differences in the total polyphenol contents among the five extracts were highly significant. The total polyphenolic content of methanolic extracts is given in Table 5.

**Figure 2:**
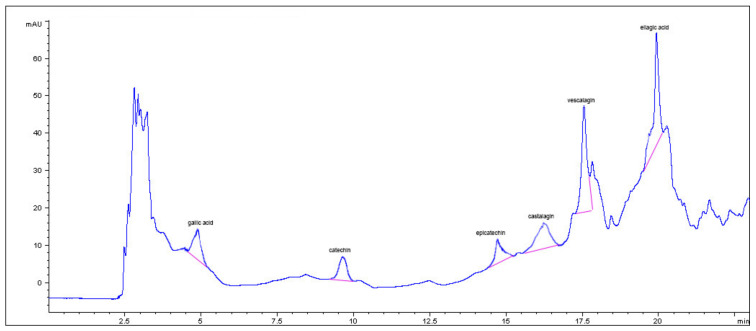
Benzoic acid derivatives, catechins, and tannins present in the different breadfruit extracts (in this case, extract ExMBA—Region of Mbadjini).

**Figure 3:**
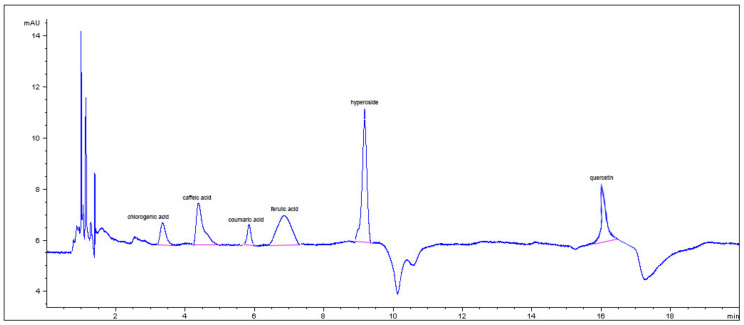
Cinnamic acid derivatives and flavonols present in the different breadfruit extracts (in this case, extract ExMBA—Region of Mbadjini).

In particular, phenolic groups identified in the five samples were derivatives of cinnamic acid, flavanol-type and flavonol-type flavonoids, benzoic acid derivatives, and tannins. The composition of the ExHAM extract is shown in Figure 2 and Figure 3 as an example of the analysed extracts.

The cultivar, origin, season, climate, and growth conditions may affect the bioactive content in the fruits. In this study, the fruit harvest period slightly varies across different sampling sites [16]. The development of tree species is also influenced by natural factors (endogenous or exogenous) as well as by human factors. These factors may influence the chemical composition of plant organs and the respective derived products [17].

In this research, the sampling area showed some differences in their pedoclimatic conditions. Indeed, each species has its requirement in terms of latitude, altitude, average annual rainfall, light availability, physic-chemical soil properties, and mean temperature. Climatic conditions present direct effects on the physiological processes and phenology of the plant (e.g., growth, fruit ripening, and flowering); thus, they may also affect the availability of essential metabolites for the biosynthesis of bioactive compounds.

### 3.2. Analysis of Bioactive Substances

#### 3.2.1. Cinnamic Acid Derivatives

Among the four cinnamic acid derivatives (caffeic, chlorogenic, coumaric, and ferulic acids) identified and quantified in all the extracts, chlorogenic acid presented the highest levels between 26.57 ± 0.31 mg/100 g DW (ExMIT) and 43.80 ± 5.43 mg/100 g DW (ExMBA). Coumaric acid showed levels ranging between 11.20 ± 0.28 (ExITS) and 11.85 ± 0.305 mg/100 g DW (ExHAM). Ferulic and caffeic acids presented maximum levels of 3.04 ± 0.39 mg/100 g DW for the former (ExITS) and 2.65 ± 1.00 mg/100 g DW for the second (ExMBA). Coumaric and ferulic acids were in trace amounts in ExMBA.

In general, the contents of these acids in the different extracts are quite similar as well as comparable with other fruits (e.g., berries with a range of 1–50 mg/100 g and apple with a range of 1–20 mg/100 g) [16,17,24]. Results are shown in Table 6.

No quantitative study of phenolic compounds in breadfruit flours has still been performed. However, these molecules exhibit several pharmacological activities. Chlorogenic acid shows anxiolytic activity which may be linked to the activation of benzodiazepine receptors (GABA receptors) [38]. Chlorogenic acid has antiviral, antibacterial, and antifungal properties with low toxicity and side effects without the appearance of microbial resistance [39,40,41]. In vitro, it inhibits the hydrolysis of potato starch [42]. It delays intestinal absorption of glucose and therefore its passage into the blood [43,44]. It exerts anti-carcinogenic effects by acting on DNA repair [45,46,47]. It also presents anti-hyperglycemic activity [48]. An in vitro study has also shown that chlorogenic acid protects against the oxidation of LDL (low-density lipoprotein), a first step in the formation of atheroma deposits [49]. Caffeic acid may protect cells against damage caused by free radicals [50]. Caffeic acid treatment has been shown to inhibit the in vitro apoptosis pathway induced by NO radicals. Caffeic acid shows anti-tumour, antiviral, anti-free radical, and anti-inflammatory properties. It has been used as a natural antioxidant to inhibit the oxidation of fish lipids in food matrices [51]. Coumaric acid presents antioxidant properties [52]. It may show a role in reducing the risk of stomach cancer by reducing the formation of carcinogenic nitrosamines [53].

#### 3.2.2. Benzoic Acid Derivatives

Benzoic acid derivatives (ellagic and gallic acids) were detected with contents ranging from 0.87 ± 0.16 mg/100 g DW (ExHAM) to 9.87 ± 0.28 mg/100 g DW (ExITS) and from 0.62 ± 0.03 mg/100 g DW (ExMIT) to 5.23 ± 0.14 mg/100 g DW (ExMBA). Significant differences existed between the contents of each of these molecules in different extracts. These values are slightly lower than other fruits such as apple and berries [17,24]. Results are shown in Table 7. 

Gallic acid exhibits activity against the HSV-2 herpes virus, reducing virus replication in a concentration-dependent manner [54]. You et al. (2010) [55] showed a decrease in the growth of pulmonary adenocarcinoma exposed to gallic acid as a function of time and dose. Gallic acid also regulates gene expression and plays a role in reducing the total concentration of lipids (cholesterol and triglyceride) [56]. Gallic acid reduces the in vitro viability of lung cancer cells in mice. A combination of this molecule with anticancer drugs such as cisplatin may be an effective treatment for this type of cancer [57].

#### 3.2.3. Catechins

In the catechin group, catechin was detected at levels ranging from 0.67 ± 0.10 mg/100 g DW (ExCEN) to 4.41 ± 0.22 mg/100 g DW (ExITS) and epicatechin from 3.15 ± 0.30 mg/100 g DW (ExMIT) to 12.95 ± 0.42 mg/100 g DW (ExITS). The highest levels of these two compounds were recorded in ExITS. There were significant differences between the contents of these substances in the different extracts. Results are shown in Table 8.

Catechins may play a role in antioxidant activity and prevention of cardiovascular disease as reported by Leverve and Weststrate (2008) [58].

#### 3.2.4. Flavonols

In the flavonol class, hyperoside and quercetin were only identified and quantified in four extracts (ExMIT, ExHAM, ExITS, and ExCEN). ExMBA did not contain this compound. Quercetin was by far the most quantitatively important flavonol with levels ranging from 14.68 ± 0.19 mg/100 g DW (ExMIT) to 29.60 ± 0.28 mg/100 g DW (ExITS). The hyperoside contents varied from 0.77 ± 0.28 mg/100 g DW (ExMIT) to 1.38 ± 0.22 mg/100 g DW (ExHAM). No flavonols were detected in ExMBA. Differences in the content of these compounds in different extracts were significant (*p* < 0.05). Results are shown in Table 9.

Daily intake of quercetin for four weeks improves blood pressure in hypertensive subjects [59]. Quercetin has also been shown to improve the health of subjects suffering from sarcoidosis, a lung chronic inflammation accompanied by oxidative stress [60]. Quercetin is also used in the treatment of inflammation associated with chronic prostatitis [61]. Quercitrin, isoquercitrin, and rutin, phenolic compounds identified in different fruits and vegetables (e.g., apple, onion, broccoli, tomato, lettuce, etc.), and well-known for their nutritional and pharmacological properties [59,62], were not detected in all the extracts. Flavonols are cardio-protectors thanks to their antioxidant activity (protection against oxidation of LDL) and the inhibition of platelet activity and their vasodilatory properties [63].

#### 3.2.5. Tannins

Tannins (castalagin and vescalagin) were detected with contents ranging from 4.76 ± 0.28 mg/100 g DW (ExHAM) to 15.66 ± 5.42 mg/100 g DW (ExMBA) for the first and from 5.97 ± 0.15 mg/100 g DW (ExMIT) to 22.38 ± 0.23 mg/100 g DW (ExHAM) for the second. Statistical analysis revealed significant differences (*p* < 0.05) between the contents of these two compounds in the different extracts. Results are shown in Table 10.

#### 3.2.6. Organic Acids

No investigation of organic acids, except ascorbic acid, has been carried out on breadfruits from different areas in the Comoros Islands in previous studies. In this study, quinic acid with contents ranging from 77.25 ± 6.04 mg/100 g DW (ExMBA) to 658.56 ± 0.25 mg/100 g DW of (ExHAM) and succinic acid with levels between 225.13 ± 0.16 mg/100 g DW (ExHAM) and 323.71 ± 0.31 mg/100 g DW (ExCEN) were the most important organic acids in all the breadfruit extracts, except ExMBA. Oxalic acid (less than 10 mg/100 g DW) was the least quantitatively important. Malic acid was not detected. Results are shown in Table 11.

Differences in organic acid levels among the different extracts varied depending on the single compound. Indeed, they were significant for specific acids such as quinic, succinic, and citric acids, but not significant for oxalic and tartaric acids. Quinic and succinic acids were distinguished from the other organic acids detected in breadfruit extracts by their significantly higher levels. For these two compounds, differences in their contents were detected among the extracts: for quinic acid, levels varied from 309.5 ± 0.44 (ExMIT) to 658.56 ± 0.25 mg/100 gDW (ExHAM) and for succinic acid from 225.13 ± 0.16 (ExHAM) to 323.71 ± 0.31 mg/100 gDW (ExCEN). Instead, the contents of the other acids in the different extracts were appreciably similar.

Organic acids are very important antioxidants with multiple uses in pharmacology [64]. Citric acid plays an important role in regulating the functioning of the urinary tract by inhibiting the adhesion of calcium oxalate crystals to renal epithelial cells [65]. The abundance of organic acids in breadfruit could play an important role in preventing some pathologies.

#### 3.2.7. Monoterpenes

Three monoterpenes (limonene, γ-terpinene, and terpinolene) were detected and identified in all the extracts, while phellandrene and sabinene were not detected. Limonene and γ-terpinene were quantitatively the main compounds in all the extracts with contents between 85.86 ± 0.23 mg/100 g DW (ExMIT) and 565.45 ± 0.24 mg/100 g DW (ExITS) for the first and between 83.51 ± 0.33/100 g DW (ExMIT) and 309.83 ±0.18 mg/100 g DW (ExITS) for the second. The highest levels were recorded in ExITS. Contents of the identified different substances are shown in Table 12.

Phellandrene and sabinene were only quantified in ExMBA with levels of 44.63 ± 4.27 mg/100 g DW and 52.98 ± 1.08 mg/100 g DW, respectively. Terpinolene contents varied between 13.23 ± 0.23 mg/100 g DW (ExITS) and 15.63 ± 0.23 mg/100 g DW (ExHAM). Differences between limonene and γ-terpinene content in the extracts were significant, while the differences were not statistically significant for terpinolene that was present in the extracts in relatively little amounts.

This research was only focused on five monoterpenes but because of the diversity of pharmacological properties of this group, it would be interesting to extend the investigations to other monoterpene compounds. Indeed, monoterpenes show antidiabetic activity [66] and therapeutic potential in the treatment of inflammatory diseases [67]. In addition, they are non-nutritive dietetic substances responsible for the antibacterial and anti-tumour activities of essential oils of several plants [68,69]. They present a chemo-preventive activity against several types of cancers [70].

#### 3.2.8. Vitamin C 

Vitamin C contents of the five extracts varied from 27.95 ± 0.04 mg/100 g DW (ExITS) and 35.40 ± 1.46 mg/100 g DW (ExMBA).

All these levels were significantly higher than those (about 23 mg/100 g) detected in breadfruit flour from Oceania reported by Christina et al. (2015) [71] but lower than those (approximately 84 mg/100 g) from Hawaii reported by Huang et al. (2000) [72]. A significant difference (*p* < 0.05) was observed between ExMBA and the other four extracts. Levels of vitamin C of the analysed extracts are reported in Table 13. 

Compared to vitamin C content of other fruits, levels were also higher than mango (17.5 mg/100 g) reported by Oliveira et al. (2009) [73], blueberries (12.60 ± 2.79 mg/100 g), and apple (3.91 ± 0.48 mg/100 g) [24]. However, they were lower than orange (71.12 ± 1.96 mg/100 g) [24]. Since the recommended daily intake of vitamin C is 60–90 mg/100 g, the consumption of dried breadfruit (equivalent to 400 g of fresh fruit) could provide about half of this requirement.

### 3.3. Antibacterial Activity

#### 3.3.1. Activities of Extracts in a Solid Medium (Solid Diffusion Method)

**Figure 4:**
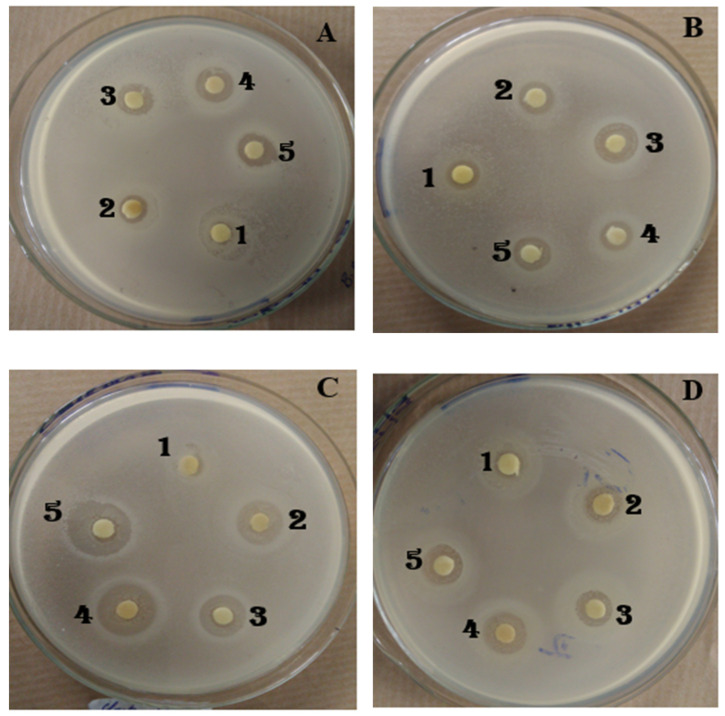
The activity of ExMIT (**1**), ExHAM (**2**), ExITS (**3**), ExCEN (**4**), and (ExMBA) (**5**) on growth in a solid medium of *Bacillus megaterium* (**A**), *Shigella flexneri* (**B**), *Clostridium perfringens* (**C**), *Listeria monoctygogenes* (**D**).

*Escherichia coli* was the only bacterial strain insensitive to all the extracts. The other 11 strains were sensitive, with the IHD ranging from 8 to 16 mm. However, the IHD depended on the bacterial strain and the used extract. *Pseudomonas aeruginosa* was resistant to three out of five extracts, and all the IHDs were less than or equal to 8 mm. *Salmonella enterica*, *Clostridium perfringens*, and *Vibrio fischeri* were found to be the most sensitive with IHDs greater than 14 mm to ExITS. Results are shown in Table 14 and Figure 4.

ExITS was the most effective among the extracts: it was active against 11 out of 12 strains. ExMIT was the relatively least effective extract with four non-susceptible strains. In all the cases, the reference antibiotic (Impenemus at 10 µg/disc) was more active than extracts.

#### 3.3.2. MIC, CMB, and CMB/MIC of the Different Extracts (Liquid Microdilution Method)

MIC values ranged from 3.12 mg/mL to 50 mg/mL. However, these values varied depending on the extract: 6.25 to 12.5 mg/mL for ExMIT, 3.12 to 12.5 mg/mL for ExHAM, 12.5 to 25 mg/mL for ExITS, 6.25 to 25 mg/mL for ExCEN, and 12.5 to 50 mg/mL for ExMBA. ExHAM was the most effective among the extracts with 58% of MICs less than 12.5 mg/mL and ExMBA the least effective with 92% of MICs greater than or equal to 25 mg/mL. *Bacillus megaterium* was the most sensitive with MICs less than or equal to 12.5 mg/mL. *Pseudomonas aeruginosa*, *Shigella flexneri*, and *Vibrio fischeri* were the least sensitive with all MICs greater than or equal to 12.5 mg/mL. Most of the CMB values were greater than 50 mg/mL, 60% of which were greater than 100 mg/mL. For *Bacillus megatorium*, *Clostridium perfringens*, and *Salmonella enterica*, 100% of the CMBs were greater than 100 mg/mL.

**Figure 5:**
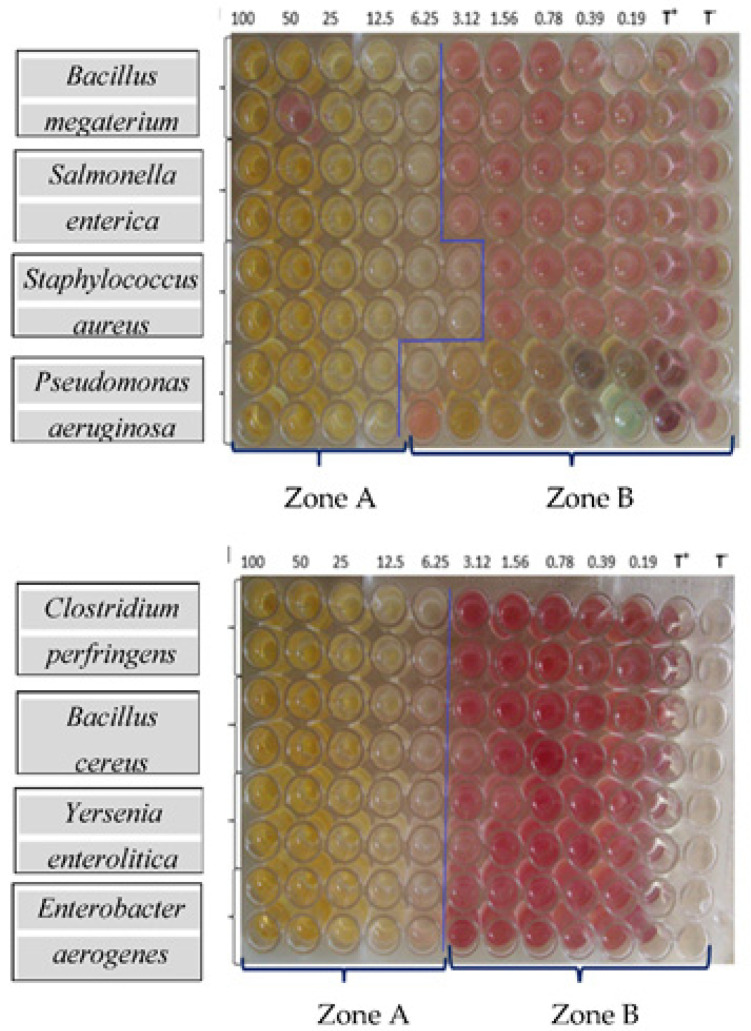

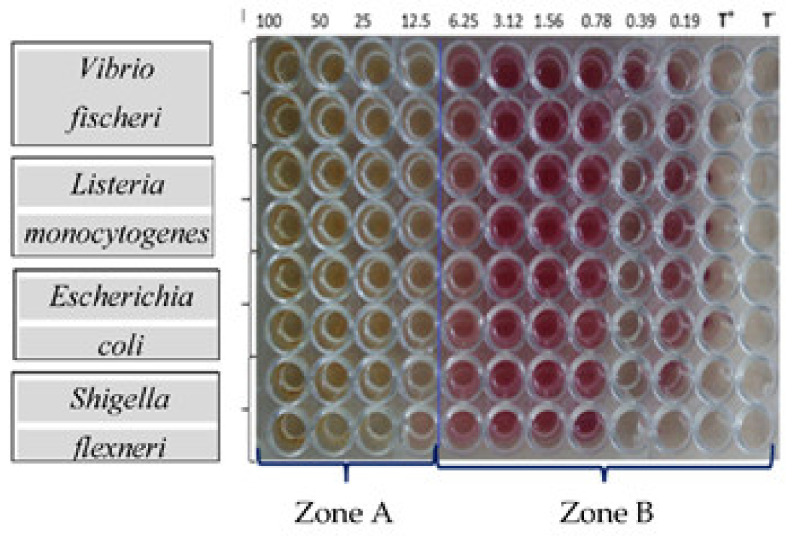
Variation of the turbidity induced by the growth of bacteria depending on the concentration of the ExHAM extract on the strains tested. ZONE A: no bacterial growth resulting in the very light yellow colour; ZONE B: visible bacterial growth resulting in purple colour; T+: negative control (100 μL of MHB + 40 μL of INT solution); T−: positive control (95 μL of MHB + 5 μL inoculum + 40 μL of INT solution).

Regarding the effects of the different extracts on tested strains, proportions of bacteriostatic and bactericidal effects varied depending on the extract. Indeed, for bactericidal effect, proportions ranged from 17% (ExMIT) to 42% (ExMBA). On *Bacillus megatorium*, *Clostridium perfringens*, *Salmonella enterica* and *Vibrio fischeri*, all the extracts showed a bacteriostatic effect. For the same sensitive strains, the number of extracts with a bactericidal effect varied from one to three: one single extract for *Enterobacter aerogenes* and three extracts for *Escherichia coli*, *Staphylococcus aureus* and *Pseudomonas aeruginosa*. Results of the MIC and CMB determination of the extracts are shown in Figure 5 and Table 15.

#### 3.3.3. Antibacterial Activity by Comparison between Solid Diffusion and Liquid Microdilution Methods

Two methods were used for the assessment of antibacterial activity (solid diffusion method and liquid microdilution method, respectively). Results obtained by these methods were not always consistent. Indeed, the best activity defined by the first method did not always match with the activity determined by the second. For example, on *Staphylococcus aureus*, ExHAM showed a low IHD (8.5 mm) and the best MIC (3.12 mg/mL), while on *Salmonella enterica*, ExITS presented the highest IHD (16 mm) and a low MIC (12.5 mg/mL). *Escherichia coli*, the only strain insensitive to all the extracts on solid medium, showed a sensitivity similar to the other strains on liquid medium. These differences between the two methods may be due to the different properties of the active ingredients in relation to the two media and/or to negative interactions between the different substances in a liquid medium.

Regarding the antibiogram method, according to the literature used for the result interpretation [31,32], ExITS was the most effective extract with 11 sensitive strains on 12 tested strains and the best IHD. In particular, with a IHD of 14.66 mm and a MIC of 1.56 mg/mL, *Shigella flexneri* was the most sensitive to the breadfruit extracts. For this reason, these extracts may be useful against shigellosis, a food and waterborne disease caused by bacteria such as *Shigella flexneri* and characterised by acute gastroenteritis; in this case, the stool is usually accompanied by blood and mucus caused by abscesses of the intestinal walls due to the invasion by these bacteria. Additionally, all the extracts showed a broad spectrum of activity as they were active against both Gram (+) and Gram (−) bacteria. This research was only a preliminary study on the potential antibacterial activity of breadfruit flours from the Comoros Islands; other bacterial strains should be used to evaluate breadfruit flours fully as reported in other similar studies [74,75]. Moreover, the same strains, sensitive to the considered extracts, should be also tested with breadfruit extracts derived from other genotypes and origins. Indeed, the tested extracts were less active than the preparations derived from Indian cultivars on *Bacillus cereus* (maximum value 10.5 ± 0.7 mm versus 16.0 ± 0.5 mm), *Staphylococcus aureus* (maximum value 10.5 ± 0.70 mm versus 20.50 ± 0.76 mm), and *Escherichia coli* (7.00 ± 0.00 mm versus 15.16 ± 0.28 mm) [75]. Additionally, extracts of Indian breadfruit flours [74], as most of the Comorian breadfruit extracts, are inactive against *Pseudomonas aeruginosa*.

The present study provided new insights on the potential antibacterial activity of the *Artocarpus altilis* fruits against different pathogenic bacteria. In the future, this research may be supplemented by tests on bacteria and fungi responsible for other serious diseases in humans (e.g., *Enterococcus faecalis*, *Streptococcus mutans*, *Candida albicans*, etc.) to evaluate their sensitivity to the *A. altilis* fruit extracts [74,75].

### 3.4. Antioxidant Capacity

The antioxidant capacity of the analysed extracts varied depending on the plant material origin. The highest value was 14.83 ± 0.11 mmol Fe^2 +^/kg (ExHAM), and the lowest value was 5.44 ± 0.35 mmol Fe^2+^/kg (ExMBA). Differences in the antioxidant capacity of the different extracts were significant (*p* < 0.05). However, some extracts showed similar results, as ExCEN and ExMBA. These results are similar to the antioxidant capacity of other common fruits as apples, berries, and chestnut as reported in previous studies [16,17,24,70]. Results obtained during this study are presented in Table 16.

The comparison between these results and literature data on other *Artocarpus altilis* cultivars or species was very difficult because the method used for the analysis (FRAP or DPPH methods), and/or the condition of the plant material (fresh or dried fruits) were often not the same. In this study, the FRAP method on the flours derived from dried fruits was utilised. The values obtained from Comorian breadfruit flours (in particular, the sample ExHAM with a mean value of 14.83 ± 0.11 mmol Fe^2+^/Kg) were similar or lower than data obtained in other studies (2015) [76] using plant material from Malaysia (22.10 ± 0.85 mmol Fe^2+^/Kg for *Artocarpus altilis*, 38.81 ± 3.01 mmol Fe^2+^/Kg for *Artocarpus integer*, and 18.10 ± 0.62 mmol Fe^2+^/Kg for *Artocarpus heterophyllus*), while they were higher than values obtained from a cultivar of *Artocarpus heterophyllus* from Uganda (1.5 ± 0.7 mmol Fe^2+^/Kg) [77].

The antioxidant compounds show many benefits for human health; in particular, the natural antioxidants are in high demand because their use should not cause potential side effects [77,78,79,80]. Most of these bioactive compounds were identified and quantified in the Comorian breadfruit extracts (e.g., phenolic compounds, flavonoids, sterols, terpenoids, and organic acids). Indeed, the breadfruit flours derived by fruits from the biggest island of the Comoros Islands showed that these bioactive substances and the differences in the antioxidant capacity between the different extracts may be mainly due to the differences between the contents of these molecules in the relative extracts.

## 4. Conclusions

This study showed that breadfruit flours from different regions of the Comoros Islands presented secondary metabolites well known for their important antibacterial and antioxidant properties. The results promote the consumption of this traditional food in the Comoros Islands as a potential health-promoting food; moreover, it may be used as a source of bioactive molecules for the food industry thanks to its antioxidant and antibacterial properties. Since these substances and their properties were not still fully explored in breadfruit fruits, this research provided new data on the potential use of these fruits as a health-promoting food. 

Significant differences in phytochemical composition and health-promoting properties were detected among fruits from different Comorian regions. For this reason, it was important to evaluate the fruits of five areas of Grande Comore in order to select different plant materials from these regions to obtain fruits with the highest contents of specific bioactive compounds. In particular, the Region of Mbadjini showed the highest content values for the main bioactive compounds.

This work was only preliminary research on the health-promoting potential of *Artocarpus altilis* fruits, and further studies on different cultivars should be performed to confirm this first hypothesis. It will be also important to (i) isolate and define the chemical structure of bioactive substances; (ii) extend antibacterial testing to other pathogens; (iii) extend the investigations to other organs of *Artocarpus altilis*; (iv) perform the same study on *Artocarpus altilis* fruits from Moheli and Anjouan, the other two islands of the Comoros Islands.

## Figures and Tables

**Table 1:** Identification code of the extracts of the fruits harvested in the five study areas.

Harvest Area	ID Extracts
Mitsamihuli	ExMIT
Hamahamet	ExHAM
Itsandra	ExITS
Centre	ExCEN
Mbadjini	ExMBA

**Table 2:** List of tested bacterial strains.

Strains	Gram Stain	References
*Bacillus cereus*	+	ATCC 14579
*Bacillus megaterium*	+	LMG 7127
*Clostridium perfringens*	+	ATCC 13124
*Listeria monocytogenes*	+	ATCC 19114
*Staphylococcus aureus*	+	ATCC 25923
*Yersinia enterolitica*	+	ATCC 23715
*Vibrio fisheri*	−	ATCC 7744
*Shigella flexneri*	−	ATCC 12022
*Pseudomonas aeruginosa*	−	ATCC 10145
*Enterobacter aerogenes*	−	ATCC 13048
*Escherichia coli*	−	ATCC 25922
*Salmonella enterica*	−	ATCC 13076

Gram-positive bacteria = “+” and Gram-negative bacteria = “−” according to the Gram’s method.

**Table 3:** Standards used for the expression of results obtained by the disk method.

IHD (x)	Results	Sensitivity of Bacteria
<8 mm	−	Insensitive
9 mm < x < 14 mm	+	Sensitive
15 mm < x < 19 mm	++	Very sensitive
x > 20 mm	+++	Extremely sensitive

**Table 4:** Results of the phytochemical screening.

Chemical Group	Test	Results				
		ExMIT	ExHAM	ExITS	ExCEN	ExMBA
Alkaloids	MAYER	−	−	−	−	−
	DRAGENDORF	−	−	−	−	−
	WAGNER	−	−	−	−	−
Deoxyosis	KELLER-KILIANI	+	+	+	+	+
Saponins	Foam index	−	−	−	−	−
Tannins and polyphenols	Gélatin 1%	+	+	+	+	+
	Salted gelatin	+	+	+	+	+
	Ferric chloride	−	−	−	−	−
Flavonoids and leucoanthocyanins	WILSTATER	+	−	−	−	−
	BATE-SMITH	+++	+++	+++	++	+++
Iridoids		−	−	−	−	−
Steroids and Triterpenes	LIEBERMANN-BURCHARD	+	++	++	++	++
	SALKOWSKI	++	+	++	++	++

not detected: −; small quantities: +; medium quantities: ++; high quantities: +++.

**Table 5:** Total polyphenol content in the different breadfruit extracts.

Extracts	Total Polyphenolic Content (TPC) (mgGAE/100 g DW)
ExCEN	35.98 ± 4.27 ^c^
ExITS	96.14 ± 2.07 ^a^
ExMIT	62.43 ± 1.76 ^b^
ExHAM	38.60 ± 0.80 ^c^
ExMBA	29.69±1.40 ^d^

Values represent the mean of three measures ± standard deviation (SD). Different letters indicate the statistically significant differences among the different extracts at *p* < 0.05. MIT: Region of Mitsamihuli; HAM: Region of Hamahamet; ITS: Region of Itsandra; CEN: Region of Centre; MBA: Region of Mbadjini.

**Table 6:** Composition and contents of cinnamic acid derivatives in the different breadfruit extracts.

Cinnamic Acids (mg/100 g DW)	ExMIT	ExHAM	ExITS	ExCEN	ExMBA
Caffeic acid	1.60 ± 0.41 ^b^	1.61 ± 0.04 ^b^	1.53 ± 0.18 ^b^	1.57 ± 0.05 ^b^	2.65 ± 1.00 ^a^
Chlorogenic acid	26.57 ± 0.31 ^c^	27.55 ± 0.28 ^b^	26.80 ± 0.23 ^c^	27.22 ± 0.23 ^b^	43.80 ± 5.43 ^a^
Coumaric acid	11.21 ± 0.18 ^a^	11.85 ± 0.30 ^a^	11.20 ± 0.28 ^a^	11.61 ± 0.26 ^a^	n.q.
Ferulic acid	1.65 ± 0.31 ^c^	1.54 ± 0.18 ^c^	3.04 ± 0.39 ^a^	2.09 ± 0.24 ^b^	n.q.

Values represent the mean of 3 measures ± standard deviation (SD). DW: dry weight; n.q.: not quantified. Different letters indicate the statistically significant differences among the different extracts at *p* < 0.05. MIT: Region of Mitsamihuli; HAM: Region of Hamahamet; ITS: Region of Itsandra; CEN: Region of Centre; MBA: Region of Mbadjini.

**Table 7:** Composition and contents of benzoic acid derivatives in the different breadfruit extracts.

Benzoic Acids (mg/100 g DW)	ExMIT	ExHAM	ExITS	ExCEN	ExMBA
Ellagic acid	4.03 ± 0.29 ^c^	0.87 ± 0.16 ^d^	9.87 ± 0.28 ^a^	1.08 ± 0.21 ^d^	5.69 ± 0.08 ^b^
Gallic acid	0.62 ± 0.03 ^d^	1.42 ± 0.26 ^c^	0.90 ± 0.09 ^d^	1.81 ± 0.07 ^b^	5.23 ± 0.14 ^a^

Values represent the mean of 3 measures ± standard deviation (SD). DW: dry weight. Different letters indicate the statistically significant differences among the different extracts at *p* < 0.05. MIT: Region of Mitsamihuli; HAM: Region of Hamahamet; ITS: Region of Itsandra; CEN: Region of Centre; MBA: Region of Mbadjini.

**Table 8:** Composition and catechin contents in the different extracts.

Extracts	Catechin (mg/100 g DW)	Epicatechin (mg/100 g DW)
ExMIT	0.95 ± 0.06 ^b^	3.15 ± 0.30 ^c^
ExHAM	1.10 ± 0.11 ^b^	3.24 ± 0.18 ^c^
ExITS	4.41 ± 0.22 ^a^	12.95 ± 0.42 ^a^
ExCEN	0.67 ± 0.10 ^c^	3.56 ± 0.39 ^c^
ExMBA	n.q.	7.76 ± 0.31 ^b^

Values represent the mean of 3 measures ± standard deviation (SD). DW: dry weight; n.q.: not quantified. Different letters indicate the statistically significant differences among the different extracts at *p* < 0.05. MIT: Region of Mitsamihuli; HAM: Region of Hamahamet; ITS: Region of Itsandra; CEN: Region of Centre; MBA: Region of Mbadjini.

**Table 9:** Composition and content of flavonols in different extracts.

Phenolic Compounds (mg/100 g DW)	ExMIT	ExHAM	ExITS	ExCEN	ExMBA
Quercetin	14.68 ± 0.19 ^b^	16.26 ± 0.32 ^b^	29.60 ± 0.28 ^a^	16.03 ± 0.29 ^b^	nd
Hyperoside	0.77 ± 0.28 ^b^	1.38 ± 0.22 ^a^	1.09 ± 0.28 ^a^	0.80 ± 0.23 ^ab^	nd

Values represent the mean of 3 measures ± standard deviation (SD). DW: dry weight. Quercitrin, isoquercitrin, and rutin were not detected in the samples. Different letters indicate the statistically significant differences among the different extracts at *p* < 0.05. MIT: Region of Mitsamihuli; HAM: Region of Hamahamet; ITS: Region of Itsandra; CEN: Region of Centre; MBA: Region of Mbadjini.

**Table 10:** Composition and content of tannins in the different extracts.

Phenolic Compounds (mg/100 g DW)	ExMIT	ExHAM	ExITS	ExCEN	ExMBA
Castalagin	9.06 ± 0.36 ^b^	4.76 ± 0.28 ^d^	6.95 ± 0.29 ^c^	5.42 ± 0.30 ^cd^	15.66 ± 5.42 ^a^
Vescalagin	5.97 ± 0.15 ^c^	22.38 ± 0.23 ^a^	10.01 ± 0.39 ^bc^	14.88 ± 0.08 ^b^	13.53 ± 4.94 ^b^

Values represent the mean of 3 measures ± standard deviation (SD). DW: dry weight. Different letters indicate the statistically significant differences among the different extracts at *p* < 0.05. MIT: Region of Mitsamihuli; HAM: Region of Hamahamet; ITS: Region of Itsandra; CEN: Region of Centre; MBA: Region of Mbadjini.

**Table 11:** Composition and organic acid content of the different extracts.

Extracts	Organic Acids (mg/100 g DW)					
	Citric ACID	Malic acid	Oxalic Acid	Quinic Acid	Succinic Acid	Tartric Acid
ExMIT	41.89 ± 0.13	n.d.	9.37 ± 0.31 ^a^	309.50 ± 0.44 ^c^	225.45 ± 0.29 ^c^	22.95 ± 0.30 ^b^
ExHAM	60.72 ± 0.28	n.d.	5.94 ± 0.38 ^b^	658.56 ± 0.25 ^a^	225.13 ± 0.16 ^c^	19.30 ± 0.34 ^b^
ExITS	52.14 ± 0.30	n.d.	9.52 ± 0.20 ^a^	633.27 ± 0.16 ^a^	283.74 ± 0.35 ^b^	31.24 ± 0.43 ^a^
ExCEN	64.15 ± 0.25	n.d.	7.77 ± 0.31 ^ab^	426.44 ± 0.16 ^b^	323.71 ± 0.31 ^a^	20.45 ± 0.16 ^b^
ExMBA	5.07 ± 0.77	n.d.	n.q.	77.25 ± 6.04 ^d^	n.d.	n.q.

Values represent the mean of 3 measures ± standard deviation (SD). DW: dry weight; n.d.: not detected; n.q.: not quantified. Different letters indicate the statistically significant differences among the different extracts at *p* < 0.05. MIT: Region of Mitsamihuli; HAM: Region of Hamahamet; ITS: Region of Itsandra; CEN: Region of Centre; MBA: Region of Mbadjini.

**Table 12:** Monoterpene composition in the different extracts.

Extraits	Monoterpenes (mg/100 g DW)				
	Limonene	Phellandrene	Sabinene	γ-Terpinene	Terpinolene
ExMIT	85.86 ± 0.23 ^d^	n.d.	n.d.	83.51 ± 0.33 ^c^	14.81 ± 0.26 ^ab^
ExHAM	233.55 ± 0.34 ^b^	n.d.	n.d.	141.24 ± 0.36 ^b^	15.63 ± 0.23 ^a^
ExITS	565.45 ± 0.24 ^a^	n.d.	n.d.	309.83 ± 0.18 ^a^	13.23 ± 0.23 ^b^
ExCEN	136.76 ± 0.23 ^c^	n.d.	n.d.	123.89 ± 0.16 ^b^	15.59 ± 0.35 ^a^
ExMBA	145.64 ± 40.78 ^c^	44.63 ± 4.27 ^a^	52.98 ± 1.08 ^a^	n.q.	n.q.

Values represent the mean of 3 measures ± standard deviation (SD). DW: dry weight; n.d.: not detected; n.q.: not quantified. Different letters indicate the statistically significant differences among the different extracts at *p* < 0.05. MIT: Region of Mitsamihuli; HAM: Region of Hamahamet; ITS: Region of Itsandra; CEN: Region of Centre; MBA: Region of Mbadjini.

**Table 13:** Vitamin C content in the different extracts.

Extraits	Vitamin C (mg/100 g DW)
ExMIT	28.08 ± 0.10 ^bc^
ExHAM	30.33 ± 0.03 ^b^
ExITS	27.95 ± 0.04 ^c^
ExCEN	28.28 ± 0.15 ^bc^
ExMBA	35.40 ± 1.46 ^a^

Values represent the mean of 3 measures ± standard deviation (SD). DW: dry weight. Different letters indicate the statistically significant differences among the different extracts at *p* < 0.05. MIT: Region of Mitsamihuli; HAM: Region of Hamahamet; ITS: Region of Itsandra; CEN: Region of Centre; MBA: Region of Mbadjini.

**Table 14:** Results of the antibacterial activity of the different extracts at 1 mg/disc on 12 bacterial strains.

Strains		Extract (1 mg/Disk)					Imepenem (10 µg)
		ExMIT	ExHAM	ExITS	ExCEN	ExMBA	
GRAM +	*Bacillus cereus*	7.5 ± 0.7	7.75 ± 1.06	10.5 ± 0.70	7 ± 0.00	8 ± 0.00	34
	*Bacillus megatorium*	10 ± 1.41	11.5 ± 2.13	12.5 ± 3.53	13 ± 4.26	13 ± 2.80	35
	*Clostridium perfringens*	9 ± 1.41	13 ± 1.41	15.5 ± 2.12	13.25 ± 2.47	13 ± 4.24	31
	*Listeria monocytogenes*	9.75 ± 0.35	9.75 ± 0.35	10.75 ± 1.06	8.75 ± 0.35	10.25 ± 1.76	30
	*Staphylococcus aureus*	8.5 ± 0.70	8.5 ± 0.70	10.5 ± 0.70	7.75 ± 0.35	8.5 ± 0.70	45
	*Yersenia enterolitica*	9.5 ± 0.70	9.5 ± 2.12	11.25 ± 2.47	10.5 ± 0.70	10.5 ± 0.00	32
GRAM −	*Enterobacter aerogenes*	11.25 ± 1.06	10.25 ± 0.36	10 ± 2.82	9 ± 1.41	10.5 ± 2.12	31
	*Escherichia coli*	7 ± 0.00	7 ± 0.00	7 ± 0.00	7 ± 0.00	7 ± 0.00	34
	*Pseudomonas aeruginosa*	7 ± 0.00	7 ± 0.00	8 ± 0.00	8 ± 0.00	7 ± 0.00	18
	*Salmonella enterica*	10.5 ± 0.70	11.25± 1.06	16 ± 1.41	12.25 ± 1.06	10.5 ± 0.70	33
	*Shigella flexneri*	8 ± 1.41	9.5 ± 0.70	12 ± 1.41	9.5 ± 0.70	10.25 ± 0.35	33
	*Vibrio fischeri*	10 ± 0.00	13.5 ± 3.53	14.50 ± 3.53	12.5 ± 6.36	12 ± 1.41	28

**Table 15:** MIC values (mg/mL), CMB (mg/mL), and CMB/MIC ratios of the different extracts on 12 bacterial strains.

Bacterial Strains		ExMIT			ExHAM			ExITS			ExCEN			ExMBA		
		MIC	CMB	CMB/MIC	MIC	CMB	CMB/MIC	MIC	CMB	CMB/MIC	MIC	CMB	CMB/MIC	MIC	CMB	CMB/MIC
GRAM +	*Bacillus cereus*	12.5	50	4	6.25	50	8	12.5	˃100	>4	6.25	50	4	50	100	2
	*Bacillus megatorium*	12.5	˃100	>4	6.25	˃100	>4	12.5	˃100	>4	12.5	˃100	>4	12.5	˃100	>4
	*Clostridium perfringens*	12.5	˃100	>4	6.25	˃100	>4	12.5	˃100	>4	12.5	˃100	>4	25	˃100	>4
	*Listeria monocytogenes*	6.25	100	8	12.5	50	4	12.5	˃100	>4	25	˃100	>4	25	100	2
	*Staphylococcus aureus*	12.5	100	8	3.12	100	>4	12.5	25	2	12.5	50	4	25	100	4
	*Yersenia enterolitica*	12.5	˃100	>4	6.25	˃100	>4	12.5	50	4	12.5	˃100	>4	50	˃100	>4
GRAM −	*Enterobacter aerogenes*	12.5	˃100	>4	6.25	50	4+	12.5	˃100	>4	12.5	˃100	>4	50	˃100	>4
	*Escherichia coli*	6.25	˃100	>4	12.5	˃100	>4	25	50	2	25	50	2	25	50	2
	*Pseudomonas aeruginosa*	12.5	50	4	12.5	50	4	12.5	100	8	12.5	˃100	>4	25	100	4
	*Salmonella enterica*	12.5	˃100	>4	6.25	˃100	>4	12.5	˃100	>4	12.5	˃100	>4	25	˃100	>4
	*Shigella flexneri*	12.5	˃100	>4	12.5	˃100	>4	25	50	2	25	100	4	50	˃100	>4
	*Vibrio fischeri*	12.5	˃100	>4	12.5	˃100	>4	12.5	100	8	25	˃100	>4	25	˃100	>4

**Table 16:** Antioxidant activity of breadfruit flour from different regions.

Extracts	Antioxidant Activity
	(mmol Fe^2+^/kg)
ExMIT	8.10 ± 0.12 ^bc^
ExHAM	14.83 ± 0.11 ^a^
ExITS	11.42 ± 0.17 ^b^
ExCEN	5.91 ± 0.22 ^c^
ExMBA	5.44 ± 0.35 ^c^

Results were reported as mean value ± SD. Different letters (from “a” to “c”) indicate the statistically significant differences among the different extracts at *p* < 0.05. MIT: Region of Mitsamihuli; HAM: Region of Hamahamet; ITS: Region of Itsandra; CEN: Region of Centre; MBA: Region of Mbadjini.

## Data Availability

Not applicable.

## Supplementary Materials

The following are available online at https://www.mdpi.com/article/10.3390/foods10092136/s1, Table S1: Chromatographic conditions of each used method.
