# Supplementary material for: Phytochemical Composition, Antibacterial Activity, and Antioxidant Properties of the Artocarpus altilis Fruits to Promote Their Consumption in the Comoros Islands as Potential Health-Promoting Food or a Source of Bioactive Molecules for the Food Industry
Source: Foods. 2021 Sep 9;10(9):2136. doi: 10.3390/foods10092136 (PMC8468414; doi:10.3390/foods10092136)
Supplement: Supplementary file 1 [file foods-10-02136-s001.zip › foods-1335027-supplementary.pdf]

Table S1. Chromatographic conditions of each used method.

Method	Class of Interest	Stationary Phase	Mobile Phase	Wavelength (*nm*)
**A**	Cinnamic acid, Flavonols	KINETEX – C18 column (4.6 × 150 mm, 5 μm)	A: 10 mM KH_2PO_4/H_3PO_4, pH = 2.8<br>B: CH_3CN	330
**B**	Benzoic acids, catechins, tannins	KINETEX – C18 column (4.6 × 150 mm, 5 μm)	A: $H_2O/CH_3OH/HCOOH$ (5:95:0.1 v/v/v), pH = 2.5<br>B: $CH_3OH/HCOOH$ (100:0.1 v/v)	280
**C**	Monoterpenes	KINETEX – C18 column (4.6 × 150 mm, 5 μm)	A: H_2O<br>B: CH_3CN	210, 220, 235, 250
**D**	Organic acids	KINETEX – C18 column (4.6 × 150 mm, 5 μm)	A: 10 mM KH_2PO_4/H_3PO_4, pH = 2.8<br>B: CH_3CN	214
**E**	Vitamins	KINETEX – C18 column (4.6 × 150 mm, 5 μm)	A: 5 mM $C_{16}H_{33}N(CH_3)_3Br$/50 mM KH_2PO_4, pH = 2.5<br>B: CH_3OH	261, 348

Elutions conditions:

Method A, gradient analysis: 5%B to 21%B in 17 min + 21%B in 3 min (2 min conditioning time); flow: 1.5 mL min^{-1}

Method B, gradient analysis: 3%B to 85%B in 22 min + 85%B in 1 min (2 min conditioning time); flow: 0.6 mL min^{-1}

Method C, gradient analysis: 30%B to 56%B in 15 min + 56%B in 2 min (3 min conditioning); flow: 1.0 mL min^{-1}

Method D, gradient analysis: 5%B to 14%B in 10 min + 14%B in 3 min (2 min conditioning time); flow: 0.6 mL min^{-1}

Method E, isocratic analysis: ratio of phase A and B: 95:5 in 10 min (5 min conditioning time); flow: 0.9 mL min^{-1}
